# The TLR4 agonist adjuvant SLA-SE promotes functional mucosal antibodies against a parenterally delivered ETEC vaccine

**DOI:** 10.1038/s41541-019-0116-6

**Published:** 2019-05-28

**Authors:** Hong Liang, David Poncet, Emilie Seydoux, Nicholas D. Rintala, Milton Maciel, Sophie Ruiz, Mark T. Orr

**Affiliations:** 1 Infectious Disease Research Institute, Seattle, WA USA; 2 Sanofi Pasteur, Marcy l’Etoile, France; 3 Henry M. Jackson Foundation for the Advancement of Military Medicine, Bethesda, MD USA; 4 Department of Microbiology and Immunology, Uniformed Services University of the Health Sciences, Bethesda, MD USA; 5 Department of Global Health, University of Washington, Seattle, WA USA

**Keywords:** Adjuvants, Gastrointestinal system, Vaccines

## Abstract

Many pathogens establish infection at mucosal surfaces such as the enteric pathogen *Enterotoxigenic E. coli* (ETEC). Thus, there is a pressing need for effective vaccination strategies that promote protective immunity at mucosal surfaces. Toll-like receptor (TLR) ligands have been extensively developed as vaccine adjuvants to promote systemic immunity, whereas attenuated bacterial toxins including cholera toxin and heat-labile toxin (LT) have initially been developed to promote mucosal immunity. Here we evaluate the ability of the TLR4 agonist second-generation lipid adjuvant formulated in a stable emulsion (SLA-SE) to augment functional mucosal antibodies elicited by intramuscular immunization with a recombinant ETEC vaccine antigen. We find that, in mice, parenterally delivered SLA-SE is at least as effective as the double-mutant LT (LTR192G/L211A, dmLT) adjuvant in promoting functional antibodies and eliciting intestinal IgA responses to the vaccine antigen. In addition, SLA-SE enhanced both the IgG2a response in the mucosa and serum, and the production of LT neutralizing serum antibodies elicited by dmLT four to eightfold. These results reveal unexpected mucosal adjuvant properties of this TLR4 agonist adjuvant when delivered intramuscularly. This may have a substantial impact on the development of vaccines against enteric and other mucosal pathogens.

## Mucosal immunity: parenteral vaccination hits the gut

Although offering great potential for generating intestinal immunity, vaccination by the oral route suffers from several barriers such as the breakdown of protein vaccines in the stomach and/or the induction of oral tolerance. To investigate whether these barriers can be circumvented, Mark T. Orr and colleagues at the Infectious Disease Research Institute use a parenteral (intramuscular) vaccination protocol in mice. Intramuscular immunization with an enterotoxigenic *E. coli* (ETEC) vaccine plus a Toll-like receptor 4 adjuvant in stable emulsion (SLA-SE) elicits a functional antibody response in both the gut and serum. Importantly, this intramuscular vaccination triggers robust production of IgA in the gut. These findings suggest that with the right adjuvant combination it might possible to generate potent protective mucosal immunity following parenteral immunization.

## Introduction

Enterotoxigenic *E. coli* (ETEC) is a noninvasive enteric pathogen, being one of the leading causes of moderate-to-severe diarrhea in children under the age of 5 in the developing world and is the leading cause of travelers’ diarrhea. The 2016 Global Burden of Disease survey attributed 23,000 worldwide deaths to ETEC-caused diarrhea in 2015.^1^ ETEC colonizes the small intestine mucosa by attaching to enterocytes via colonization factor (CFs) adhesins, which is an important virulence determinant. The most prevalent CFs are CFA/I and surface antigens 1–6 (CS1–CS6).^2^ ETEC causes disease by the production of heat-stable and/or heat-labile enterotoxins (ST and LT, respectively). Both toxins cause an ion imbalance, leading to a cholera-like watery diarrhea.^3^

Substantial evidence supports that the development of a prophylactic vaccine against ETEC should be feasible. In fact, prior exposure to ETEC may provide highly significant protection against reinfection with the homologous strain or with an ETEC strain expressing the same or related CFs as demonstrated in animal models and in human volunteers.^4,5^ Moreover, the cholera vaccine Dukoral^®^ (Valneva) provides short-term protection against LT-expressing ETEC by eliciting cholera toxin (CT)-specific antibodies that are cross-reactive to LT.^6^ Finally, oral passive immunization studies with antibodies directed against the colonization factor CFA/I or the tip adhesin of CFA/I, CfaE, were protective against the CFA/I+ ETEC strain H10407 in a controlled human challenge trial.^5,7,8^ These human challenge studies are especially important for ETEC vaccine development as the pathogenicity and specificity of ETEC strains differ between hosts (i.e., human and porcine ETEC strains express different CFs).

Despite these observations suggesting that an ETEC vaccine is feasible, eliciting protective antibodies against mucosal pathogens such as ETEC has proven more difficult than for many systemic infections because the most common routes of immunization, including intramuscular and subcutaneous, are relatively ineffective in eliciting mucosal antibody responses. This is especially true for subunit vaccines, such as recombinant proteins, that lack innate immune stimulatory properties found in attenuated or inactivated whole pathogen vaccines. Mucosal delivery of vaccines is more effective for eliciting mucosal centered immune responses, however, oral vaccination against enteric pathogens is complicated by the acidic stomach environment and the potential for inducing tolerance to the vaccine antigen by the oral route. Bacterial products such as CT and ETEC LT toxins, which share a common A–B_5_ structure, are potent mucosal adjuvants that can stimulate antigen-specific IgA responses. However, the clinical usage of this class of adjuvants has been hampered by the severe adverse events associated with intranasal delivery of wild-type LT or a single-mutant LT adjuvant (LTK63).^9,10^ A double mutant LT (LTR192G/L211A, dmLT) has been extensively investigated in the recent years and shown positive safety record and retained mucosal adjuvant properties when given orally or parenterally.^11^ We and others are investigating the use of dmLT for the parenteral delivery of ETEC subunit vaccines, where, in addition to be an adjuvant, dmLT can also elicit a protective anti-toxin response. In addition, there is the need to increase the arsenal of adjuvants that can elicit mucosal responses when used parenterally.

The second-generation lipid adjuvant (SLA) is a synthetic hexa-acylated lipid that has been optimized for activation of the human TLR4/MD2 receptor complex and builds on previous work on the synthetic TLR4 agonist glucopyranosyl lipid A (GLA).^12^ When formulated with an oil-in-water stable emulsion (SE), SLA-SE safely and potently augments systemic antibody responses and TH1 immunity in mice and humans. In mice, SLA-SE promotes class switching to IgG2 and correspondingly to IgG1 in humans.^12–15^ This preferential class-switching may be important for an ETEC vaccine as these isotypes preferentially interact with activating FcƴRs and are associated with more potent toxin neutralization and functional antibodies against a number of pathogens.^16–18^ To determine whether this adjuvant would augment the immunity of a parenterally delivered subunit ETEC vaccine, we compared the capacity of the adjuvants SLA-SE and dmLT to augment the systemic and intestinal antibody responses elicited by a recombinant antigen, CfaEB consisting of the minor and major subunits of the CFA/I fimbriae.^19^ Furthermore, we evaluated dmLT as a vaccine antigen in addition to its adjuvant properties, as up to 50% of disease caused by ETEC are attributed to LT-expressing strains of the bacteria, with or without co-expression of ST.^2^ We also evaluated the potential synergistic benefits of including both adjuvants for improving both CFA/I and LT directed immunity.

## Results

### SLA-SE augments mucosal antibodies to a parenteral immunization

**Fig. 1:**
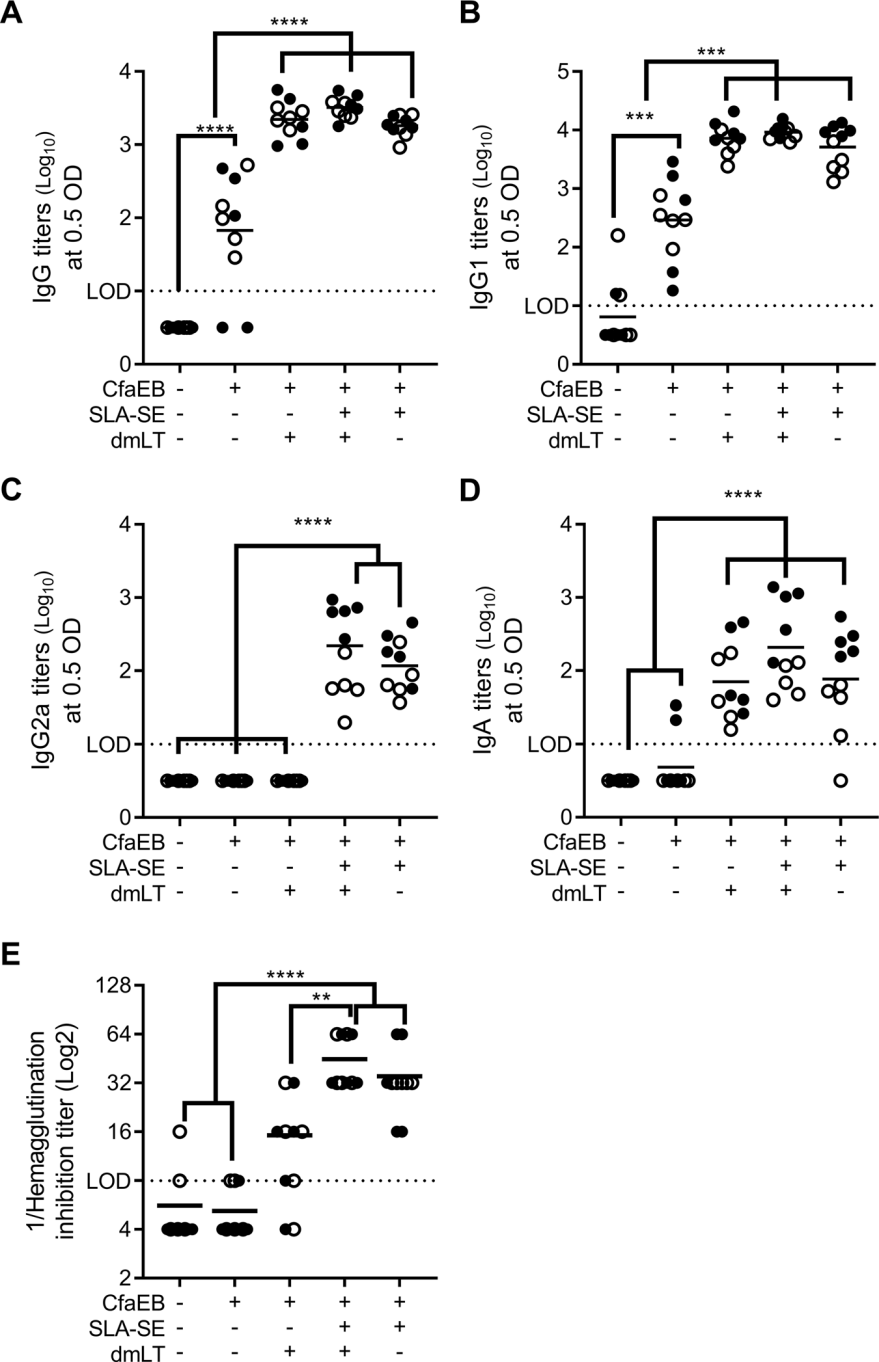
SLA-SE augments mucosal antibody responses against a parenterally delivered vaccine antigen CfaEB. BALB/c (*n* = 5 per group, per experiment) were immunized twice on Days 0 and 21 via intramuscular immunization with CfaEB +/− SLA-SE and/or dmLT. Intestinal wash samples were collected on Day 35 and assessed for CfaEB-specific IgG (**a**), IgG1 (**b**), IgG2a (**c**), or IgA (**d**) by ELISA and log_10_ transformed. **e** Functional anti-CFA/I antibody titers from the intestinal wash samples were determined by HAI with human red blood cells using ETEC strain H10407. Horizontal bars are drawn at the mean. The lower limit of detection (LOD) is indicated by the dashed line. Data pooled from two experiments indicated with open and closed symbols, respectively, with similar results. *, **, ***, and **** indicate *P* < 0.05, 0.01, 0.001, and 0.0001, respectively, as determined by one-way ANOVA using Tukey’s multiple comparisons test. All other pairwise comparisons between the groups were not significantly different

To determine whether a parenterally delivered TLR4 agonist adjuvant can elicit a similar mucosal immune response as an A-B_5_ class of adjuvant, BALB/c mice were immunized intramuscularly with an ETEC candidate vaccine antigen, CfaEB, adjuvanted with dmLT, SLA–SE or both. Following two homologous intramuscular (IM) immunizations, intestinal wash (IW) fluid was collected to obtain mucosal antibodies. Parenteral immunization with the protein alone produced low levels of anti-CfaEB IgG responses in the IW, primarily of the IgG1 subclass (Fig. 1a–c). SLA-SE augmented this response to a level similar to that achieved with dmLT; there was no further enhancement of IgG or IgG1 subclass responses when the two adjuvants were combined. However, unlike dmLT, SLA-SE significantly enhanced mucosal anti-CfaEB IgG2a levels (Fig. 1c). Of interest, even though dmLT alone did not augment IgG2 titers, these responses increased when dmLT was combined with SLA-SE. Notably, dmLT and SLA-SE each augmented the IgA response to CfaEB and demonstrated some additive benefit when both adjuvants were included (Fig. 1d).

Parenteral immunization with recombinant antigen alone did not elicit functional mucosal antibody titers as determined by hemagglutination inhibition (HAI) with the CFA/I+ ETEC strain H10407 (Fig. 1e). As with IgG2a titers, dmLT did not augment the functional neutralizing antibody response. Conversely, SLA-SE significantly augmented the functional mucosal antibody response, which was not further augmented by the presence of dmLT as a co-adjuvant (Fig. 1e). Taken together these data demonstrate that inclusion of a TLR4 agonist adjuvant such as SLA-SE is at least as effective as the mucosal adjuvant dmLT in eliciting intestinal antibody responses to a parenteral immunization, especially IgA and functional antibodies.

**Fig. 2:**
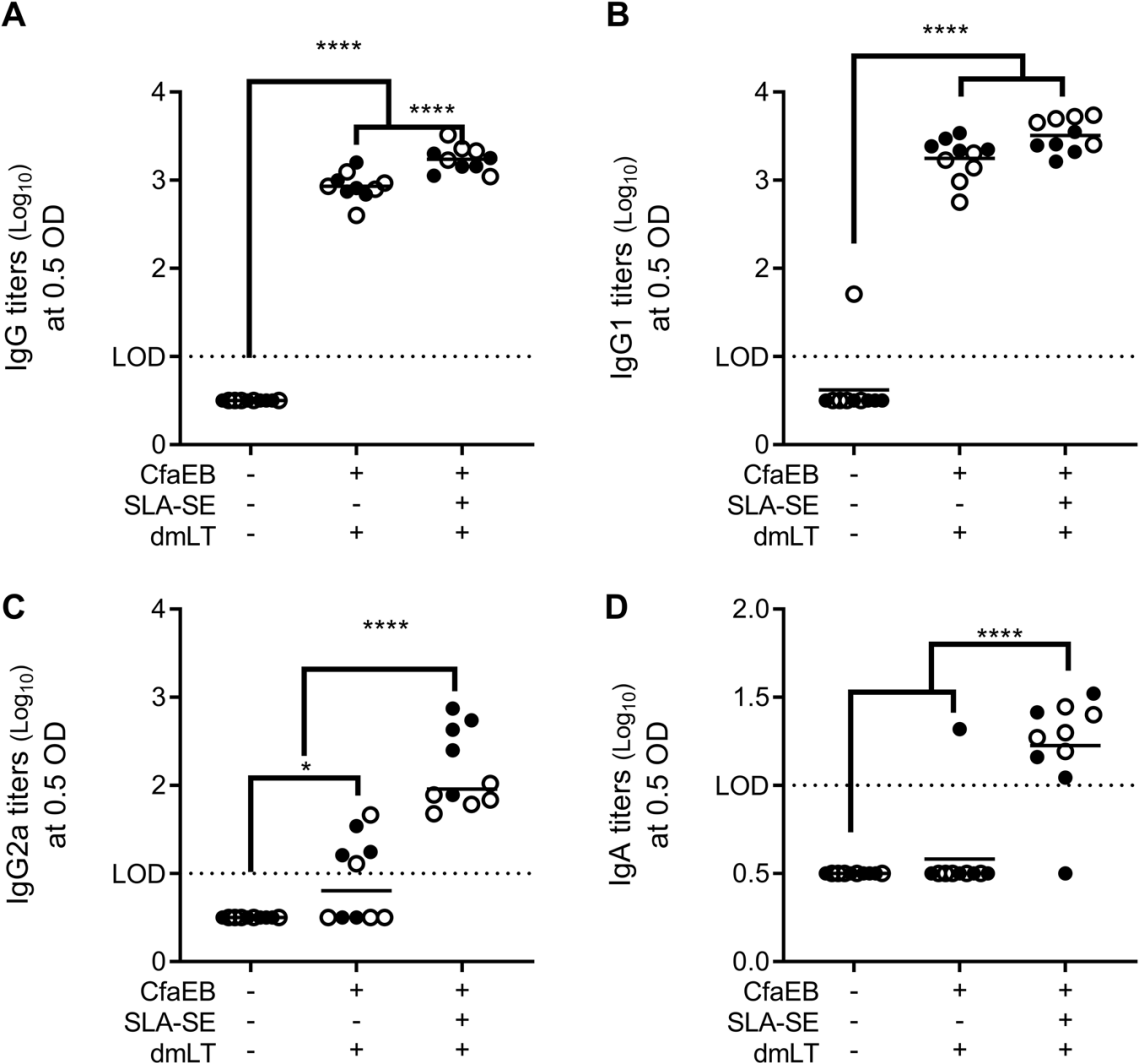
SLA-SE augments mucosal antibody responses against dmLT. BALB/c (*n* = 5 per group, per experiment) were immunized as in Fig. 1. Intestinal wash samples were collected on Day 35 and assessed for dmLT-specific IgG (**a**), IgG1 (**b**), IgG2a (**c**), or IgA (**d**) by ELISA and log_10_ transformed. Horizontal bars are drawn at the mean. The lower limit of detection (LOD) is indicated by the dashed line. Data pooled from two experiments indicated with open and closed symbols, respectively, with similar results. *, and **** indicate *P* < 0.05 and 0.0001, respectively, as determined by one-way ANOVA using Tukey’s multiple comparisons test. All other pairwise comparisons between the groups were not significantly different

Since dmLT is also an antigen able to generate anti-toxin response, we also evaluated whether inclusion of SLA-SE augmented anti-dmLT immunity in the IW. SLA-SE augmented the dmLT-specific total IgG titers, but not the IgG1 subclass (Fig. 2a, b). Addition of SLA-SE shifted the IgG response to dmLT from an IgG1-only response to a balanced IgG1/IgG2 response (Fig. 2c). Most importantly, for a vaccine directed against an intestinal pathogen, such as ETEC, inclusion of SLA-SE was necessary to produce a measurable dmLT-specific IgA response with the IM delivery route of this dmLT dose (Fig. 2d). Functional LT-neutralizing antibodies were not detectable in the IW fluid, regardless of immunization.

### SLA-SE augments functional antibody responses in the serum

**Fig. 3:**
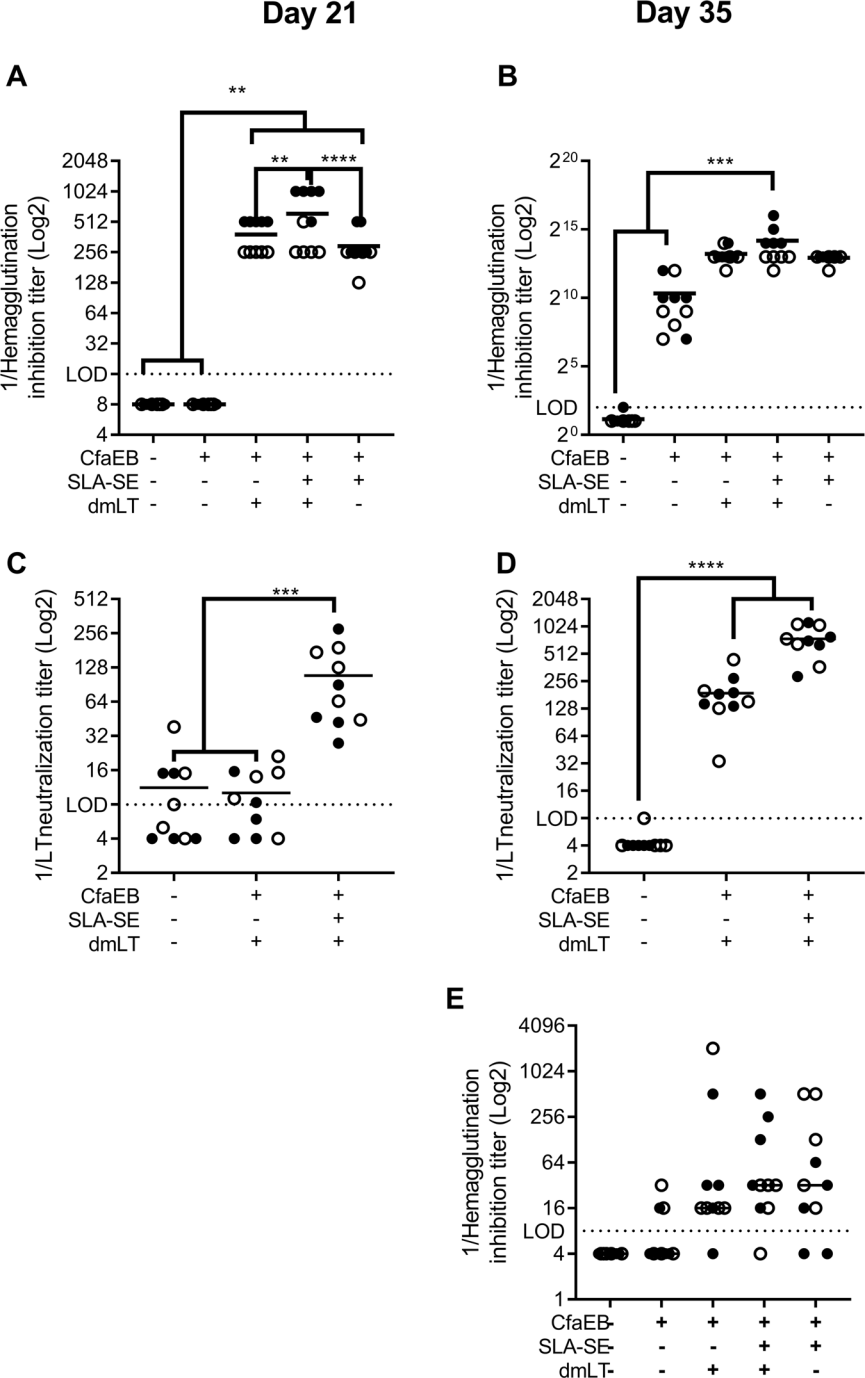
SLA-SE augments functional systemic serum antibodies against CFA/I and LT. BALB/c (*n* = 5 per group, per experiment) were immunized as in Fig. 1. Serum samples were collected on Day 21 after the priming immunization (**a**, **c**) and 14 days after the booster immunization (**b**, **d**, **e**). Functional anti-CFA/I antibody titers from the intestinal wash samples were determined by HAI against human red blood cells using ETEC strain H10407 (**a**, **b**) or WS3294A (**e**). **c**, **d** LT-neutralizing antibodies were determined by inhibition of cAMP flux in Vero cells treated with LT toxin. Horizontal bars are drawn at the geometric mean. The lower limit of detection (LOD) is indicated by the dashed line. Data pooled from two experiments indicated with open and closed symbols, respectively, with similar results. **, ***, and **** indicate *P* < 0.01, 0.001, and 0.0001, respectively, as determined by one-way ANOVA using Tukey’s multiple comparisons test. All other pairwise comparisons between the groups were not significantly different

Circulating antibodies may also transudate from the blood into the mucosal tissue where they can provide protection.^20,21^ Therefore, we assessed the functional serum antibodies elicited after the initial and booster immunization. Two IM immunizations were required to produce functional HAI titers with the unadjuvanted CfaEB antigen (Fig. 3a, b). Inclusion of either dmLT or SLA-SE promoted significantly higher HAI responses after the priming immunization, which was further augmented with the booster immunization. SLA-SE and dmLT provided similar levels of enhancement of HAI titers. The combination of dmLT and SLA-SE produced an additional significant increase in HAI titers (Fig. 3a). CFA/I and CS14 are closely related colonization factors, thus there would be a considerable benefit to vaccine coverage if a CFA/I derived antigen could elicit anti-CS14 functional antibodies.^22^ Immunization with CfaEB alone was not sufficient to produce significant HAI titers against the CS14+ ETEC strain WS3294A. However, addition of dmLT and/or SLA-SE together did produce a robust HAI titer against this heterologous ETEC strain, suggesting that one benefit of these adjuvants may be to increase protective immunity against nonvaccine bacterial strains (Fig. 3e).

Antibodies raised against dmLT are able to block the activity of the LT toxin, as determined by neutralization of LT elicited cAMP flux.^3^ However, we found that two immunizations of dmLT were required to produce significant LT neutralizing titers in the serum (Fig. 3c, d). Addition of SLA-SE enhanced a functional anti-LT response after the priming immunization and further increased this response after the booster immunization. Taken together these data indicate that addition of SLA-SE promotes functional circulating antibodies against both CfaEB and LT of a potential ETEC vaccine.

### SLA-SE promotes serum IgG2 titers

**Fig. 4:**
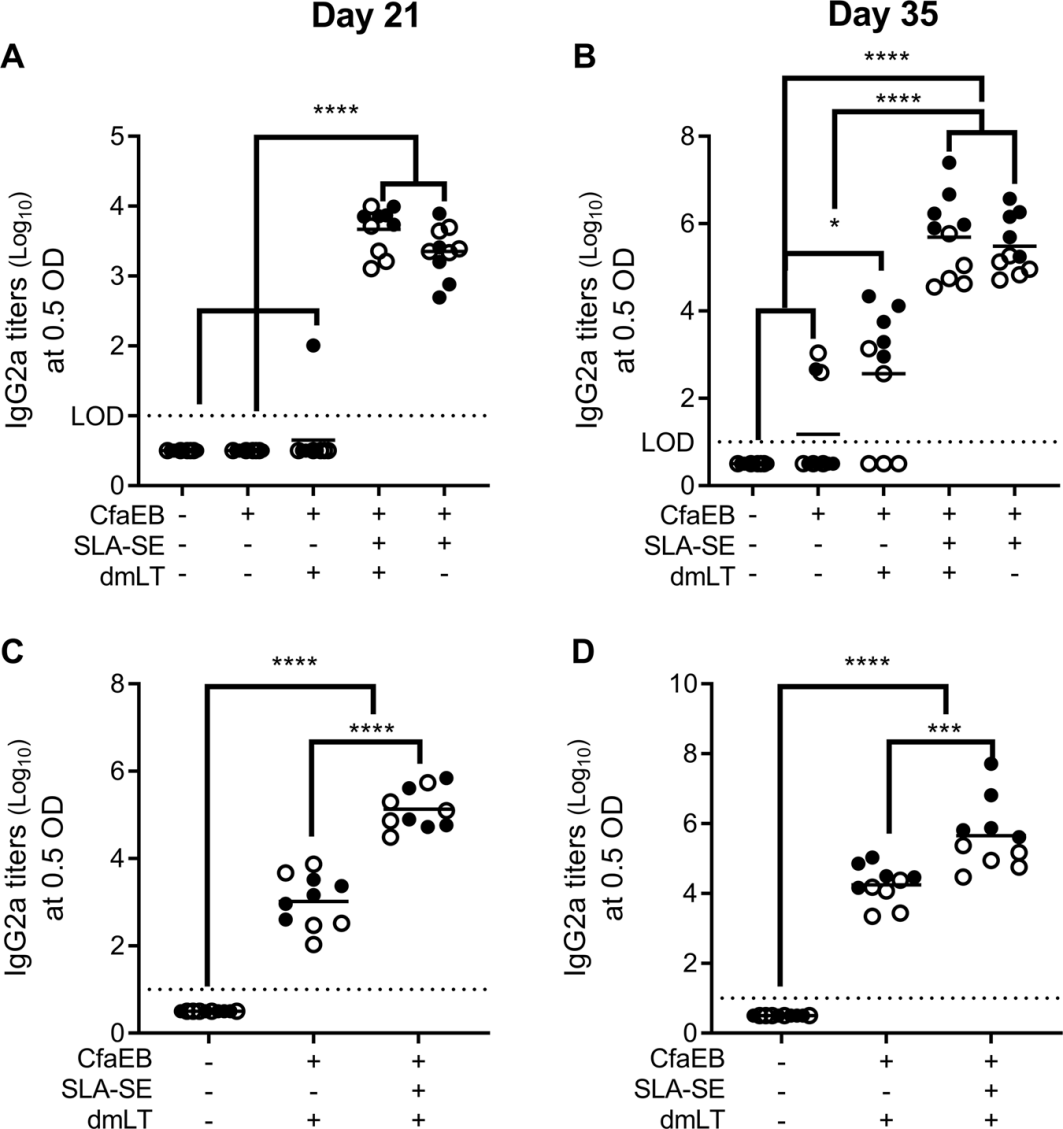
SLA-SE augments systemic serum IgG2 responses to CfaEB and dmLT. BALB/c (*n* = 5 per group, per experiment) were immunized as in Fig. 1. Serum samples were collected on Day 21 after the priming immunization (**a**, **c**) and 14 days after the booster immunization (**b**, **d**). Serum samples assessed for CfaEB-specific (**a**, **b**) or dmLT-specific IgG2a (**c**, **d**) by ELISA and log_10_ transformed. **e** Horizontal bars are drawn at the mean. The lower limit of detection (LOD) is indicated by the dashed line. Data pooled from two experiments indicated with open and closed symbols, respectively, with similar results. *, **, ***, and **** indicate *P* < 0.05, 0.01, 0.001, and 0.0001, respectively, as determined by one-way ANOVA using Tukey’s multiple comparisons test. All other pairwise comparisons between the groups were not significantly different

**Fig. 5:**
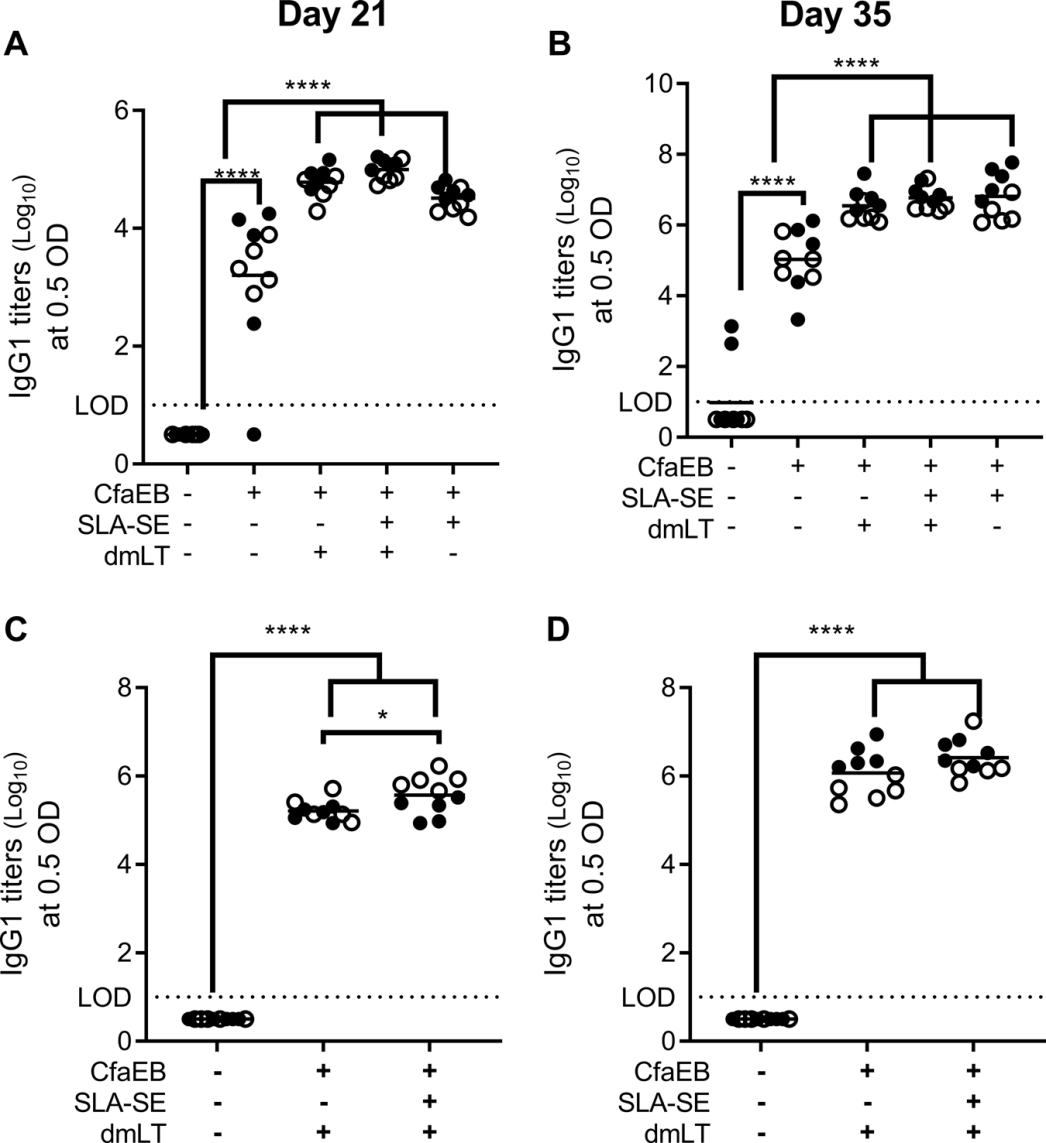
SLA-SE augments systemic serum IgG1 responses to CfaEB and dmLT. BALB/c (*n* = 5 per group, per experiment) were immunized as in Fig. 1. Serum samples were collected **a**, **c** on day 21 after the priming immunization and **b**, **d** 14 days after the booster immunization. Serum samples assessed for **a**, **b** CfaEB-specific or **c**, **d** dmLT-specific IgG1 by ELISA and log_10_ transformed. Horizontal bars are drawn at the mean. The lower limit of detection (LOD) is indicated by the dashed line. Data pooled from two experiments indicated with open and closed symbols, respectively, with similar results. *, **** indicate *P* < 0.05 and 0.0001, respectively, as determined by one-way ANOVA using Tukey’s multiple comparisons test. All other pairwise comparisons between the groups were not significantly different

Similar to the IW samples, addition of SLA-SE to CfaEB+dmLT augmented the IgG2a responses to both antigens after both the prime and boost immunizations more effectively than dmLT (Fig. 4). Conversely, immunization with CfaEB alone produced robust IgG1 response after the prime, which was further augmented with the booster immunization (Fig. 5a, b). Similarly, after the priming immunization with dmLT there were high dmLT-specific IgG1 responses. Addition of SLA-SE did not alter the IgG1 response to dmLT or CfaEB at prime or boost compared with CfaEB+dmLT immunization (Fig. 5c, d). Thus, the primary different in dmLT and SLA-SE adjuvant activity for systemic antibody responses is the promoting of IgG2 class switching.

## Discussion

Eliciting mucosal immunity, including intestinal resident IgA and transudated IgG, via parenteral immunization is a significant hurdle in the development of vaccines against enteric pathogens such as ETEC and other pathogens that preferentially infect mucosal tissue.^23,24^ Although mucosal delivery (e.g., intranasal or oral) has the potential to overcome this challenge, this approach faces challenges of its own. Intranasal delivery of vaccines adjuvanted with wild type or an attenuated LT adjuvant, LTK63, produced a number of cases of Bell’s palsy, a type of facial paralysis.^9,10^ Oral delivery of protein antigens is complicated by the acidic stomach environment, which may quickly degrade the vaccine components. Further oral delivery of vaccine antigens must overcome the induction of oral tolerance.^24^ Thus, the development of parenteral vaccination mechanisms that produce mucosal immunity would be a significant advance for the development of enteric vaccines.

We demonstrated that the synthetic TLR4 ligand SLA formulated in SE was at least as potent as dmLT in augmenting serum and intestinal IgG and IgA responses against the recombinant ETEC vaccine antigen CfaEB at the doses tested. SLA-SE was more effective than dmLT in augmenting the serum and intestinal IgG2 responses to this antigen. IgG2 responses are of particular significance for vaccine development in small animal models as it has the highest affinity for the Fcγ receptors that mediate antibody-dependent cellular cytotoxicity and phagocytosis (ADCC and ADCP).^16–18^ In agreement with this, immunizations that produced the highest CfaEB-specific IgA and IgG2a responses showed a trend towards producing the greatest CFA/I-specific HAI titers. Therefore, adjuvants such as SLA-SE that promote serum IgG2a class-switching in mice may be particularly beneficial for producing antibodies that are able to block either adherence via colonization factors or cAMP flux caused by LT and/or ST.

At the tested doses SLA-SE and dmLT were able to synergize to further augment some of the CfaEB-specific immune responses in the intestine and serum, and importantly did not show any interference compared with the single adjuvant treatments. Perhaps most surprisingly SLA-SE was even more effective in augmenting anti-LT responses than anti-CfaEB responses, despite dmLT having intrinsic adjuvant properties itself. Specifically, inclusion of SLA-SE was necessary to elicit measurable dmLT-specific IgA and IgG2a antibodies in the intestine and functional anti-LT antibodies in the serum after the first immunization. dmLT-specific serum IgG2a antibodies and functional anti-LT antibodies were further boosted after the second immunization by inclusion of SLA-SE. The reasons for SLA-SE being more beneficial for the dmLT-driven response than the CfaEB-driven response are unclear but we speculate that it may be due to the dose of dmLT being ~tenfold lower than that of CfaEB. In addition, the presence of anti-dmLT antibodies after the priming immunization may have reduced its adjuvant activity upon the booster by rapidly clearing the protein adjuvant or neutralizing its adjuvant activity; thus, inclusion of SLA-SE was necessary to overcome this limitation. Further studies will be necessary to determine if parenteral delivery of SLA-SE is able to elicit mucosal immunity in humans as it did in the mice used in the present study. Similarly, it will be important to translate the current findings of synergies between SLA and dmLT from the mouse models used here to human volunteers. Relevant to this we observed a strong correlation between the mucosal and systemic HAI responses (Spearman nonparametric correlation of 0.74 at day 21 and 0.80 at day 35 in the serum), indicating that serum sampling is a good proxy for the mucosal functional antibody responses programmed by these adjuvants, at least in the mouse system.

We have previously found that IM immunization with a recombinant tuberculosis vaccine antigen adjuvanted with the synthetic TLR4 agonist adjuvant GLA-SE augmented TH1 responses in the lungs of mice.^25^ However, these cells were almost exclusively located in the lung vasculature and did not home to the parenchyma. Despite this, parenteral immunization was sufficient to provide protection against an aerosol challenge with *M. tuberculosis*. Intranasal delivery of the same TLR4 agonist adjuvanted vaccine augmented the CD4 response in the lung parenchyma, indicating that mucosal delivery of TLR4 elicits a different quality of immunity than parenteral immunization. Conversely, IM immunization of women with a recombinant HIV antigen adjuvanted with an aqueous formulation of GLA (GLA-AF) was more effective in promoting serum and mucosal antibodies than intravaginal immunization with the antigen or intranasal immunization with the antigen adjuvanted with chitosan.^26^ Combined with the results presented here, these findings suggest that inclusion of a TLR4 agonist adjuvant may be an effective method of eliciting functional mucosal antibodies with a parenteral vaccination strategy. These findings may have broad applications for a number of mucosally targeting vaccines including those for enterics, sexually transmitted diseases, and pulmonary infections.

## Methods

### Mice, immunizations, and sample collection

Female BALB/c mice aged 6–10 weeks were purchased from the Jackson Laboratory and maintained in Specific Pathogen Free conditions. All animal experiments and protocols used in this study were approved by the Infectious Disease Research Institute’s IACUC. Cohorts of 5 mice per group were immunized twice, 3 weeks apart via an IM injection in the calf muscles of hind limb with 6.3 µg of His6-tagged CfaEB recombinant protein with or without 5 µg of SLA formulated in 2% stable emulsion and/or 0.4 µg dmLT in 100 µL total volume/dose. Antigen and adjuvant doses were selected from our previous published and unpublished works.^12,27^ Peripheral blood was collected on Day 21 by retro-orbital bleed. Central blood was collected on Day 35 by cardiac puncture from mice under deep anesthesia. Serum was separated from whole blood by centrifugation at 10,000 rpm for 5 min. ~15 cm of intestine were collected and washed with 0.5 mL of cold phosphate buffered saline (PBS). The intestinal wash (IW) was centrifuged to remove fecal matter and stored at −70 °C until use. Each experiment was repeated once and both data sets are presented.

### Antibody ELISAs

Antibody titers against CfaEB and dmLT were determined by ELISA. Corning 3700 384-well microtiter plates were coated with 2 µg/mL of CfaEB antigen or 1 µg/mL dmLT, and blocked with PBS (pH 7.1), 0.1% Tween-20, and 1% skim milk. 12-point 3- or 4-fold serial dilutions of the serum and IW samples were then carried out. Detection antibodies included IgG1, IgG2a, total IgG, and IgA (all conjugated to horse radish peroxidase [HRP]) (Southern Biotech). The plates were incubated in the HRP substrate 3,3′,5,5′-Tetramethylbenzidine (TMB) for 4 min, and the reaction was stopped using 1 N H_2_SO_4_. Finally, optical density (OD) readings were taken at 450 nm, with data reduction at 570 nm using an automated plate-reader (Biotek Synergy 2). The endpoint titers were determined using the dilution at OD = 0.5.

### Hemagglutination inhibition assay

ETEC strains H10407 (CFA/I+, LT+, STh+, STp+) and WS3924A (CS14+ STh+) were passaged and expanded on CFA agar plates supplemented with 50 µM desferal with or without 1.5 g/L bile salts. An aliquot was plated at 0.1 OD and grown to lawns for 16 h at 37 °C. The lawns were scraped into 20 mL PBS, washed twice (centrifuged at 3500 RPM for 10 min at 4 °C), resuspended in PBS + 0.5% D-mannose, and adjusted to OD_600_ = 20. Alternatively, bacteria were stored at OD_600_ = 20 in PBS + 20% glycerol + 0.5% d-mannose and protease inhibitors at −80 °C until assay.

The concentration of bacteria used in the HAI was determined by finding the minimal hemagglutination titer (MHT), the lowest concentration of bacteria needed to agglutinate the red blood cells. The MHT was determined by performing a 12-point 2-fold serial dilution of OD_600_ = 20 ETEC across one row of a 96-well U-bottom tissue culture plate. 25 µl PBS + 0.5% D-mannose and 25 µl 1.5% red blood cells were then added to each well, and the plate was then placed on a shaker at 4 °C at 450 RPM for 30 min and then 550 RPM for 1 h. The MHT is the last well of the serial dilution where the concentration of bacteria is sufficient to result in hemagglutination of red blood cells (pellet formation).

Serum and IW samples were diluted in PBS + 0.5% d-mannose in the first column of a 96-well U-bottom tissue culture plate (Falcon 353077), and each sample was serially diluted 2-fold across 12 points. 25 µl of ETEC at 4x the MHT was added to each well and incubated at 37 °C with gentle agitation for 30 min. 25 µl of 1.5% human red blood cells, collected from healthy donors and diluted in PBS, were then added to each well, followed by incubation at 4 °C on a shaker at 450 RPM for 30 min and then 550 RPM for 1 h. The HAI titer is the reciprocal of the last well to show complete clearing (absence of a pellet) caused by inhibition of hemagglutination by the antibodies present in the sample.

### LT neutralization assay

Vero cells (ATCC, #atcc-ccl-81) were maintained in IMDM complete media (IMDM + GlutaMax (Gibco, #31980-030), 4% fetal bovine serum (Sigma) and 1% penicillin–streptomycin (Sigma)) in a 37 °C, 5% CO_2_ incubator. 750 cells/well were plated in Culture Plate 384 (Perkin Elmer, 6007689) and incubated at 37 °C, 5% CO_2_ incubator for 20–24 h to allow for cells to adhere. Serum or IW samples were 12-point serial diluted in LT buffer in 384-well plate using Nimbus robot liquid handler (Hamilton). LT at 0.3 ng/µL (2×EC_10_) was then added to each dilution at equal volume. Plates were incubated at 37 °C for 15 min in shaker incubator at 100 rpm to allow antibody binding to LT. The serum+LT mixture was then added to the Vero cells using the Nimbus robot liquid handler. The cell plates were incubated for 2.5 h in 37 °C, 5% CO_2_ incubator. At the end of the incubation, 5 µL each of Eu-cAMP and uLight-anti-cAMP (Lance Ultra cAMP kit; Perkin Elmer) diluted in Detection buffer at 1:50 and 1:150, respectively, was added to each well in the cell plate, which were incubated in the dark at room temperature for 1 h. Plates were read at 665 and 615 nm using the Victor plate reader (Perkin Elmer). The 665/615 nm was determined for each sample and plotted as a function of serum dilution. The IC_50_ was calculated using Prism dose–response-inhibition, log(inhibitor) vs. response–variable slope (four parameters).

### Statistical analysis

Data were analyzed using GraphPad Prism 7 software (La Jolla, CA, USA) by one-way ANOVA (with Tukey’s multiple comparisons post-test). Values were considered significantly different with *P* *<* 0.05 (*), *P* < 0.01 (**), *P* < 0.001 (***), or *P* < 0.0001 (****).

## Data Availability

Data for all figures are available upon reasonable request to the corresponding author.
